# Social benefits and individual costs of creativity in art and science: A statistical analysis based on a theoretical framework

**DOI:** 10.1371/journal.pone.0265446

**Published:** 2022-04-27

**Authors:** Fabio Zagonari, Elena Giacomoni

**Affiliations:** 1 Dipartimento di Scienze per la Qualità della Vita, Università di Bologna, Rimini, Italy; 2 Libera Accademia di Belle Arti di Brescia, Rimini, Italy; Iowa State University, UNITED STATES

## Abstract

In this study, we statistically identified and characterized the relationship between the long-run social benefits of creativity and the in-life individual costs (in terms of happiness and health) of creativity. To do so, we referred to a theoretical framework that depicts a creator’s life. We generated a balanced dataset of 200 creators (i.e., composers, painters, mathematicians and physicists, and biologists and chemists born between 1770 and 1879), and calculated standardized evaluations of the long-run social benefits in different domains (performances, exhibitions, citations). We performed regression analysis and identified the statistical determinants of the relationship between a creator’s social benefits and the costs to their happiness and health. We found that creativity represented an individual cost for all four creator groups, with a larger impact on happiness than on health; the cost was greater if creativity was based more on divergent than on convergent thinking or if authors faced greater language issues. The impacts of long-run social benefits on individual happiness and health were similar in the arts and sciences if institutional differences were taken into account.

## Introduction

“What the common herd best appreciates is the work of artisans, the ultimate varnish which makes each work trivial and non-artistic. It is the pleasure to add truth and knowledge which makes me endeavor to finish a painting”

—Paul Cezanne

Recent neuroscience results based on functional magnetic resonance imaging have empirically identified two main determinants of creativity from an individual perspective: divergent thinking [1] and convergent thinking [2]. In this paper, we have chosen the terminology *convergent thinking* to represent the goal of finding a correct solution to a problem by following a particular set of logical steps (i.e., it resembles an artisan’s activity), whereas *divergent thinking* represents the goal of generating creative new ideas by exploring many possible solutions (i.e., it resembles a creator’s activity). The ideas and information obtained from divergent thinking can be organized and structured using convergent thinking.

To the best of our knowledge, only Zagonari [3] has numerically compared how improvements in convergent thinking and in divergent thinking produce happiness from creativity over an individual’s whole life. Once divergent thinking skill has increased sufficiently for a creator to perceive a sufficient range of creative elements, convergent thinking becomes more important for achieving happiness from creativity; that is, too much divergent thinking can decrease happiness. Consider the psychological distress and social isolation that accompany a situation in which divergent thinking seemed to prevail over convergent thinking (e.g., in many of Van Gogh’s “self-portraits” as well as in his last painting, “Melancholia”). Consider the psychological stability and social acceptability that accompany a situation in which convergent thinking seemed to prevail over divergent thinking (e.g., Schoenberg’s Suite Op. 25 based on dodecaphony in 1923 and his subsequent masterpieces). However, the impacts of creative activity on health have not been considered from the perspectives of psychological and somatic effects, and the arts have not been compared with the sciences in terms of the effects of their institutions.

From a social perspective, creativity has been defined as a novel and valuable contribution to a particular domain [4, 5]. The degree and extent of novelty and value are negotiated among participants in a specific domain (e.g., music, painting, theoretical sciences such as mathematics and physics, applied sciences such as biology and chemistry), since they depend on existing knowledge and institutionalized rules of collaboration and evaluation. Consider the roles of music and painting critics (i.e., the gatekeepers) and of the general population (i.e., the target audience) for arts, and consider the roles of peer-scientific reviewers (i.e., the gatekeepers) and the scientific community (i.e., the target audience) for sciences. A short-run evaluation (i.e., by the creator’s contemporary generation in the general population or the scientific community) might reveal different results from a long-run evaluation (i.e., by subsequent generations).

To our knowledge, only Liu et al. [6] have statistically compared contemporary evaluations (i.e., careers) in the arts and sciences within a theoretical framework based on “hot streaks”. In particular, a hot streak (i.e., a peak work or performance that leads to subsequent successful works or performances due to increased reputation and recognition) that results in a unique or innovative creation appears to occur randomly within a career (independently of the productivity timing and shorter than the career length). However, the lasting impacts of works (i.e., the long-run social benefits of creativity) and the origins of hot streaks (e.g., creativity arising from divergent thinking) have been neglected by researchers.

The purpose of the present study was to statistically identify and characterize the relationship between the costs of creativity to an individual (in terms of both happiness and health) and the long-run social benefits of creativity (in terms of specific evaluations and institutions in different domains). Note that we have chosen the words *benefits* and *costs* instead of positive and negative impacts in order to test whether creators exhibit heroic behavior (i.e., positive impacts for the whole society at the cost of negative impacts for the individual creator), although quantifying the total benefits for society is beyond the scope of this study. In this context, social benefits are *theoretically* grounded in the economic literature for composers and painters (i.e., the willingness to pay for a performance or an exhibition ticket is at least equal to the benefits people expect to receive from attending the performance or exhibition) and on the scientific literature for scientists (i.e., subsequent scientists find benefits in a creator’s research and cite these creators), although non-use values and distributional issues are disregarded. In contrast, individual costs are *empirically* estimated from an original dataset, without *a priori* positive or negative signs attached to the empirical variables that represent the costs, although these variables are based on a theoretical framework. To accomplish this goal, we refer to the analytical framework developed in Zagonari [7] that depicts a creator’s life, by producing an original dataset (i.e., the data used in the present study has not been previously used) of 200 creators (i.e., 50 composers, 50 painters, 50 mathematicians or physicists, and 50 biologists or chemists). We chose the period between 1770, Beethoven’s birth year, and 1879, Einstein’s birth year. This let us obtain relatively consistent estimates of health and happiness for each creator. Because this period ends more than 100 years ago, we believe that the creators whose works are still being performed, viewed, or cited represent long-run contributions to society (i.e., social benefits). Note that these long-run social benefits should be distinguished from the total social benefits, since the total benefits should consider all affected people from the time when a work was created to the present day for each creation, by taking into account the impacts of institutions on value formation over time. We then obtained standardized evaluations of the long-run social benefits in the different domains (i.e., average number of global performances, viewings, or citations of a creator’s works from 2009 to 2019, expressed as a percentage of the performances of Beethoven’s works, as a percentage of the number of Van Gogh’s paintings exhibited in the world’s 10 most popular museums during this period, and as a percentage of the number of citations of the works of Poisson, Einstein, Darwin, and Gibbs to represent mathematics, physics, biology and chemistry, respectively). We conclude by performing graphical regression analysis and identifying linear statistical determinants of the relationship between a creator’s long-run social impacts (i.e., over periods from around 200 to around 100 years) and the creator’s happiness and health. This let us highlight differences and similarities among the creators’ domains.

Note that we have not included interdisciplinary research, which we define as research conducted across scientific disciplines or across artistic and scientific domains [8], since such work has been relatively scarce [9] or has been unreliably evaluated [10]; it has also been rarely funded [11]. However, Zeng et al. [12] have recently shown that success in science (in terms of the average citations per paper) is negatively correlated with writing about diverse topics. Moreover, we have not included the impacts of different research groups or art movements [13] for individual successful works. Finally, we will focus on overall success in life, since the timing and productivity during a creator’s career do not affect the long-run success of a creator’s works [14]. However, Yin et al. [15] have recently shown that ultimate success in science, which they defined based on funded grant applications submitted to the National Institutes of Health in the U.S., depends on the dynamics of past successes and failures.

## Materials and methods

Any statistical analysis of the relationship between social benefits and individual costs in terms of health (HEA) and happiness (HAP) requires consistent data for all creators in the dataset. In this section, we will describe how we met this requirement by empirically estimating a theoretical model based on an original dataset.

### The mathematical model

Zagonari [7] represented the dynamic interrelationship between happiness (*hap*[*t*]) and health (*hea*[*t*]) at each time *t* by using two dynamic equations for an individual’s achievements (*y*[*t*]), in which standardizations are applied to the original family income *fy* and to the individual’s original health *fh*, while parameters are represented by the reference group’s average achievement *ay*, the education level *ed*, the feasible set for opportunities *os*, the ethical freedom *fr*, the number of past periods that affect the current health *me*, the occupation type *oc*, and the employment status *em*:

hap[t]=α{(y[t]–fs)/fs}+β{(y[t]–y[t–1])/y[t–1]}+γ{(y[t]–ay)/ay}+hea[t] (1)

$$hea\left[t\right]=os+{\sum_{t-me}}^{t-1}hap\left[t\right]–\delta \left(y\left[t\right]–y\left[t–1\right]\right)+y\left[t\right]+em+ed+oc$$ (2)

where:

$$\begin{array}{c} \alpha =1\;\left(\text{Aristotle}\right)\;\text{or}\;\beta =1\;\left(\text{Epicurus}\right)\;\text{or}\;\gamma =1\;\left(\text{Zeno}\right)\\ fs=fs\left[t\right]–fs\left[t–1\right]=fy+fh–u\left[t\right]+fr;\delta \geq 0,oc\leq 0,em\geq 0,me\geq 1;\;\text{and}\;u\left[t\right]\;\text{is}\;\text{in}\;\left[–u*,+u*\right] \end{array}$$

where α represents Aristotle’s contribution to happiness (achievements with respect to the individual’s opportunity set), β represents Epicurus’ contribution (short-run achievements), γ represents Zeno’s contribution (achievements with respect to the individual’s reference group), such that α + β + γ = 1, *u*[*t*] is the personal uncertainty, δ depicts the level of psychological stress due to missed achievements, and u* is the long-run equilibrium uncertainty. Note that we set all coefficients at 1 to simplify the notation: their values could instead be obtained by statistical analyses. Moreover, units for α, β and γ are happiness over the specific contribution to HAP they refer to, whereas the unit for δ is health over its specific contribution to HEA. Finally, we will refer to Eqs 1 and 2 as “the life model”, by using capital letters to stress that we are moving from a theoretical to an empirical model.

The previous paragraphs highlighted which data are theoretically required at the individual’s level to estimate the life model. Hereafter, we refer to the domains of creative endeavor as CO = composers of music, PA = painters, MP = mathematicians or physicists to represent more theoretical scientists, and BC = biologists or chemists to represent more applied scientists. Supplementary Materials I in S1 Text presents the complete list of empirical variables used in our analysis. To produce a balanced sample, we chose 50 creators for CO, 50 creators for PA, 50 creators for MP, and 50 creators for BC (see Supplementary Materials II in S1 Text for the complete list of creators). Note that we chose creators who could be ranked among the best 50 in their domain based on criteria specified below, who were approximately equal notable, and for whom approximately the same level of detail was available for their lives. For each creator, we recorded the birth year (BY), the death year (DY), and consequently the life years (LY) = DY–BY. Note that these data and all individual data for each creator detailed below were obtained by reading a total of 200 biographies and by coding the values of each parameter based on the information provided in the biography according to objective and quantitative criteria specified below. We have made the coding data available via the OSF repository under reference number https://osf.io/qz73t. We analyzed composers (born between 1770 and 1879) whose compositions were performed from 2009 to 2019 at a significant level (i.e., at a rate equal to at least 1% of the number of Beethoven performances) around the world. We obtained this data from bachtrack.com for concerts and operabase.com for operas. Note that we were not forced to exclude many composers (e.g., De Falla, Glazunov, Rode, Spontini) to limit the number of composers to 50 because we could not obtain a balanced sample of 100 creators in each of the four domains (i.e., we limited our sample to 50 creators in each domain because the domain with the fewest creators (i.e., composers) contained only 50 members who met our criteria). In other words, to limit our sample to 50 composers, we included only the 50 composers who were performed most often between 2009 and 2019.

Fraiberger et al. [16] studied artist exhibitions, including auction sales and primary market quotes for around 500,000 artists. To select only 50 painters so that our sample for each domain would be balanced, we considered only painters born between 1770 and 1879 whose paintings are exhibited at a significant level (i.e., at 1% or more of the number of van Gogh paintings in the same museums) in the world’s 10 most popular museums (based on the number of visitors per year, www.theartnewspaper.com): the Louvre in Paris, the Metropolitan Museum of Art in New York, the British Museum in London, the Tate Modern in London, the National Gallery in London, the National Gallery in Washington, the Musée D’Orsay in Paris, the Victoria and Albert in London, the Museo Nacional Del Prado in Madrid, and the Hermitage in St. Petersburg. Note that these museums provide a good representation of painters from the period under consideration, although the oldest painters in the sample such as Klee or Kandinsky and subsequent painters are inevitably under-represented in these museums.

We analyzed the main scientists in the four disciplines (two theoretical vs. two applied) by counting the number of citations of each scientist’s work, supplemented by including Nobel winners in Physics and Chemistry from 1901 (the first year the prize was awarded) to 1921 (the year of Einstein’s award). Note that we did not account for the creators’ production over their whole life (i.e., total number of compositions, paintings, or articles), since this was not relevant for successful works in the CO, MP, and BC categories (i.e., a single work can be performed many times in many places in the world, in general, and in particular, consider one-opera composers such as Leoncavallo, with his opera “Pagliacci”, or Mascagni, with his opera “Cavalleria Rusticana”). However, such lifetime production may be significant for PA (i.e., a given painting cannot be exhibited simultaneously in two or more museums), in general, and for the very productive Van Gogh, in particular. However, the outstanding productivity of Van Gogh does not affect our results, since we used him as the reference painter to calculate the long-run social benefits.

For health, the goal is to depict the potential impacts of artistic and scientific activities on the individual’s health to represent the three main dynamic determinants of health (i.e., genetics, chance, and behavior). We neglected accidents or illness (e.g., pneumonia, cholera, typhus, syphilis, tuberculosis, meningitis). To do so, we included genetics in the health status (HS) (i.e., reducing the HS value of 3 assigned to each creator as good health status by 1 point for creators with diseases such as chronic nephritis, osteogenesis imperfecta, and chronic granulomatous: thus HS becomes 2 as medium health status) and included behaviors that could be described as psychological problems (PP; i.e., reducing HS values by 1 in the case of PP such as depression, hypersensitivity, paranoia, obsessive-compulsive disorder, bipolar disorder, nervous breakdown) and somatic problems (SP; i.e., reducing HS by 1 in the case of SP such as heart attack, cancers (e.g., throat, kidney, pancreatic, lung, colon), endocarditis, brain aneurysm, diabetes, liver cirrhosis).

We defined the employment status (EM) as 1 if the creator was employed at a conservatory, academy, or university, but as 0 otherwise. Note that Borodin was employed as a doctor, so for him, EM = 0. In other words, we assumed that a creator who worked in a day job that did not focus on their creative work was not employed.

For the Aristotle component of happiness, to which we applied the weight α, we set the economic status of the creator’s birth family (FS) and the creator’s economic status (CS) at 1 = poor, 2 = medium, and 3 = rich, where FS or CS = 1 for a creator’s parents or creators who were primary or secondary school professors or retail dealers, 2 if they were elected or appointed as government officials and chair professors at a conservatory, academy, or university, and 3 if they were lawyers, doctors, traders, land owners, or business owners.

We set the marital status (MA) to 1 if the creator was legally married. Note that Tchaikovsky got married to cover his homosexuality, but we treated this as MA = 1.

For the Epicurus component of happiness, to which we applied the weight β, we set the year of their first personal success (SY) to be the year of the first successful composition for composers and painting for painters and as the year of the first appointment as a full professor or chair professor at a university for the two science categories. We used this to calculate the number of years of successful professional life (i.e., DY–SY).

For the Zeno component of happiness, to which we assigned a weight of γ, we chose the year of the first social endorsement (AY) as the year when the creator received their first social award (e.g., the Legion of Honor in France, appointment to the Royal Society in Britain, Nobel prize) for all four groups of creators. This let us calculate the number of years of successful social life (i.e., DY–AY). Note that we treated the Nobel prize as a social award rather than as a professional award for two reasons: it cannot be used as a primary criterion for professional achievement, since otherwise the vast majority of scientists would not have achieved a successful “professional” life, and it was introduced in 1901, so it cannot be used for the scientists from previous periods in our sample.

Table 1 presents the main descriptive statistics for our sample of creators. For each statistic, we tested for significant differences between pairs of creator groups using Student’s *t* test with a threshold value of 1.290 (i.e., *P* < 0.1 with 98 degrees of freedoms). Two main conclusions can be obtained. First, since the results for many of the features were expected, our sample appears to be trustworthy. In particular:

- The time lag between the creator’s birth and their success (i.e., SY–BY) were similar for the four domains. This insight is consistent with Liu et al. [6].
- The economic status of the creator’s birth family (FS) differed, as expected: PA > MP > BC > CO. Consider, for example, that students who start painting because it increases their health and happiness status and who continue painting because they can afford to do so (e.g., Toulouse-Lautrec).
- The creator’s economic status (CS) differed among the groups, as expected: BC > CO ~ MP = PA. Consider, for example, the money obtained by exploiting scientific breakthroughs in biology or chemistry (e.g., Baekeland).
- The marital status (MA) differed among the groups, as expected: BC > MP > CO > PA (reflecting different life styles of creators in different domains during the period under consideration).
- The employment status (EM) differed among the groups, as expected: MP = BC > CO > PA. Consider that university employment would be the main source of money for mathematicians and physicists.
- Success rates, which equal (DY–SY)/LY, as a %), were greater for the two art groups than for the two science groups, as expected: CO > PA > MP = BC. A chair position at a university at a success rate of 74% is reasonable; however, unsuccessful CO included Borodin, Fauré, and Mussorgsky and unsuccessful PA included Gauguin, Van Gogh, and Vuillard.
- Gain rates, which equal (CS–FS)/FS, as a %), differed among the groups, as expected: CO > BC > PA = MP. This might be due to the lowest economic status of the creator’s birth family (FS) for CO, and the highest creator’s economic status (CS) for BC.
- Loss rates, which equaled (FS–CS)/FS, as a %), differed among the groups, as expected: PA = MP > CO = BC. This might be due to the highest economic status of the creator’s birth family (FS) for PA and MP, the lowest economic status of the creator’s birth family (FS) for CO, and the highest creator’s economic status (CS) for BC.
- Award rates, which equaled (DY–AY)/DY, as a %), differed among the groups, as expected: BC > MP > CO > PA. This might be due to a greater social endorsement for applied than for theoretical science, and for music than for painting in the period under consideration.
- Original health statuses (i.e., HS in [1,3], HS at 3 as a %, and HS at 2 as a %) were similar for the four domains: CO = BC ≈ PA > MP. Nobody had HS = 1.
- Psychological problems (i.e., PP at 1, as a %) differed among the groups, as expected: CO > PA > MP = BC. Nobody had PP = 2 or 3.
- Somatic problems (i.e., SP at 1, as a %) differed among the groups, as expected: CO > PA > MP = BC. Nobody had SP = 2 or 3.

**Table 1:** The descriptive statistics for the sample of 200 creators.

Factors	CO	PA	MP	BC	CO ≠ PA	CO ≠ MP	CO ≠ BC	PA ≠ MP	PA ≠ BC	MP ≠ BC
	Statistics for each group				*t*-test values for comparisons between groups					
LY (years): Mean	62	65	67	71	-0.55	-0.99	-1.54	-0.44	-0.98	-0.54
Success lag (i.e., SY-BY, years): Mean	36	36	36	37	0.25	-0.08	-0.14	-0.34	-0.40	-0.06
FS in [1, 3]: Mean	1.68	2.12	2.04	1.96	**-2.47**	**-2.08**	**-1.64**	0.42	0.84	0.43
CS in [1, 3]: Mean	2.04	2.02	2.02	2.16	0.11	0.11	-0.62	0.00	-0.73	-0.74
HS in [1, 3]: Mean	2.86	2.84	2.78	2.86	0.08	0.33	-0.01	0.25	-0.09	-0.34
MA (%)	0.64	0.48	0.72	0.70	**2.28**	-1.05	-0.80	**-3.40**	-3.13	0.26
EM (%)	0.66	0.18	0.84	0.84	**8.04**	**-2.33**	**-2.33**	**-10.29**	**-10.29**	0.00
Success rate (i.e., SY < DY, %)	0.98	0.98	0.74	0.74	0.00	**2.95**	**2.95**	**2.95**	**2.95**	0.00
Gain rate (i.e., CS > FS, %)	0.40	0.18	0.18	0.26	**4.30**	**4.30**	**2.57**	0.00	**-1.77**	**-1.77**
Loss rate (i.e., CS < FS, %)	0.10	0.22	0.22	0.10	**-3.09**	**-3.09**	0.00	0.00	**3.09**	**3.09**
Award rate (i.e., AY > 0, %)	0.48	0.22	0.60	0.76	**4.67**	**-1.77**	**-3.92**	**-6.39**	**-8.48**	**-2.15**
Good health status (i.e., HS = 3, %)	0.86	0.84	0.78	0.88	0.08	0.33	-0.08	0.25	-0.16	-0.41
Medium health status (i.e., HS = 2, %)	0.14	0.16	0.22	0.14	-0.27	-0.97	0.00	-0.71	0.27	0.97
Psychological problems (PP = 1, %)	0.26	0.16	0.04	0.04	**2.26**	**5.90**	**5.92**	**3.89**	**3.92**	0.04
Somatic problems (SP = 1, %)	0.36	0.24	0.1	0.1	**2.30**	**5.68**	**5.72**	**3.51**	**3.56**	0.06

Abbreviations: LY = life years, SY = success year, BY = birth year, FS = birth family economic status in [1, 3], DY = death year, CS = creator economic status, MA = married, EM = employed, AY = award year, HS = health status in [1, 3], PP = psychological problems in [1, 3], and SP = somatic problems in [1, 3]. Creator groups: CO = composers of music, PA = painters, MP = mathematicians or physicists, and BC = biologists or chemists. Notes: % values are expressed as decimals; comparisons between creator groups are the values of Student’s t test, with a threshold value of 1.290 for significance and statistically significant values boldfaced.

Notes: HS = 2 for CO (Albeniz, Berlioz, Chopin, Elgar, Mendelssohn, Schubert, Johann Strauss), for PA (Friedrich, Manet, Matisse, Ranson, Renoir, Toulouse-Lautrec, Troyon, Van Gogh), for MP (Cantor, Carnot, Clausius, Hamilton, Hermite, Hertz, Stark, Maxwell, Rayleigh, Riemann), and for BC (Bunsen, Fisher, Hess, Mendel, Mendeleev, Pasteur, Sklodowska). PP = 1 for CO (Beethoven, Bizet, Bruckner, Chopin, Donizetti, Mendelssohn, Mussorgsky, Reger, Rossini, Schumann, Smetana, Tchaikovsky, Wagner), for PA (Cézanne, Constable, Friedrich, Gauguin, Gericault, Toulouse-Lautrec, Troyon, Van Gogh), for MP (Boltzmann, Cantor), and for BC (Bosch, Schleiden). SP = 1 for CO (Bellini, Bizet, Borodin, Brahms, Busoni, Debussy, Donizetti, Elgar, Grieg, Mahler, Massenet, Paganini, Puccini, Rachmaninoff, Reger, Respighi, Rimskij-Korsakov, Rossini), for PA (Cezanne, Church, Constable, Courbet, Degas, Matisse, Monet, Renoir, Sargent, Seurat, Signac, Sisley), for MP (Hamilton, Hertz, Maxwell, Millikan, Poincaré), and for BC (Cvet, Haber, Koch, Mendel, Nobel).

Note that the lowest value of the economic status of the creator’s birth family (FS) for CO is plausible, since working in an orchestra was an option for young students of music, whereas the lowest value of the creator’s economic status (CS) for PA is reasonable, since teaching in schools was not an option for unsuccessful painters. Moreover, the life years (LY) for data from all creator groups combined increased slowly but significantly with increasing birth year (BY) (the slope of the linear regression was 0.068 with *t* at 1.97 and *P* < 0.05), which represents an increase in life expectancy of around 25 days per year during the period under consideration. The life years (LY) also increased significantly with increasing health status (HS) (the slope of the linear regression was 2.558 with *t* at 4.97 and *P* < 0.01), which highlights the consistency of the health data for each creator. In particular, the life years (LY) was greater for painters than for composers, but shorter in these groups than in the two science groups, and was shorter for theoretical scientists than for applied scientists [17]. Finally, the health status (HS) did not change significantly during the study period for data from all creators combined (i.e., the birth year (BY) was positively but not significantly related to health status (HS), with a slope of 0.003 and with *t* at 0.64 and *P* > 0.52), which excludes both a lack of information about older creators and an impact of medicine advances in the period under consideration. Thus, our sample does not seem to be affected by significant spurious correlations.

Second, since many features were common between art and science (i.e., it was not possible to group composers and painters on the one side and theoretic and applied scientists on the other side), the statistical analyses of our sample must be developed by considering all four domains (i.e., CO, PA, MP, and BC) instead of two domains (i.e., CO and PA in art, MP and BC in science). Note that the vast majority of artists and scientists were male in our sample, which prevented us from testing for a significant impact of gender on creativity, happiness, or health (e.g., [18]).

### The empirical model

Because we could not obtain data for all creators on the parameter values in the life model for each year *t* in the study period, it was necessary to estimate Eqs 1 and 2 at the creator’s end of life (year *T*) by implementing the following three steps:

*First*, since the health status (HS) takes values from 1 to 3 and we have information on psychological problems and somatic problems for a creator’s whole life, we evaluated HEA at death by applying the following formula:

HEA[T]=HS(1–0.33PP)(1–0.33SP)/3

Note that our choice of a scale from 1 to 3 will not affect the results, since it will be included in the regression intercepts rather than in the regression slopes. In other words, we measured HEA[*T*] as the health status (HS) reduced by possible psychological and somatic problems suffered during a creator’s life.

*Second*, since both psychological and somatic problems affect life quality by about 25% [19–22], we calculated HAP[*T*] by applying the following formula:

HAP[T]=0.25{α(CS–FS)/FS+β(DY–SY)/LY+γ(DY–AY)/LY}+0.75HEA[T] (3)

Note that a change in HEA by 1 unit due to either a psychological or a somatic problem (e.g., from 3 to 2) will affect HAP by 0.25 (i.e., 0.33 × 0.75). In other words, we measured happiness as 0.25 times satisfaction (i.e., the three sources of happiness, namely α × an increase in socioeconomic status with respect to the family status expressed as a percentage of FS, β × years of professional success as a percentage of LY, and γ × years of social success as a percentage of LY) plus 0.75 times health. We did this to make the scale used for health status consistent with the impact of psychological or somatic problems on the quality of life. Moreover, we assumed β* = 0.75 as the average *empirical* value in [0.5, 1], since the Epicurus component (i.e., short-run achievements) is likely to be the most prominent happiness component for the creators (i.e., β* ≥ 0.5): later in this section we test this parameter’s value in terms of the overall strength (i.e., *R*^2^) of the regressions of HEA as a function of HAP, the economic status of the creator’s birth family (FS), the creator’s economic status (CS), CO, PA, MP, and BC. Finally, we assumed α* = γ* = 0.125 (i.e., the three weights sum to 1 when β* = 0.75, and we assigned them the same value because we had no reason to differentiate between them), since the Aristotle and Zeno components are likely to be the least prominent happiness components for the creators (indeed, creators are almost always the first creator in their birth family and they are very often an outstanding creator within their reference group).

*Third*, we estimated the following empirical version of Eq (2):

HEA[T]=HAP[T]+FS+CS+OC+ε (4)

where ε represents the stochastic error term and *T* is the year at a creator’s end of life. Note that we approximated the creator’s education level using the economic status of the creator’s birth family (FS) as a proxy, under the assumption that the ability of creators to obtain an overall education depended on the socioeconomic status of their original family, and we assumed that this was in addition to a good education in their own creator domain. In contrast, we neglected the employment status (EM) in Eq 4 based on the collected data, since the vast majority of the authors were employed at a conservatory, academy, or university. Indeed, the overall strength of the model (i.e., *R*^2^) was not affected by introducing EM (see Supplementary Materials IV in S1 Text). However, we will use EM to represent a social cost and an individual support for people taking up a life of creative exploration (see the Results). Moreover, we treated the occupation type OC as dummy variables (i.e., CO = 1 if the creator is a composer, PA = 1 if the creator is a painter, MP = 1 if the creator is a mathematician or a physicist, and BC = 1 if the creator is a biologist or a chemist). Finally, we disregarded personal uncertainty due to a lack of data, disregarded the psychological stress due to missed achievements (i.e., δ) because we focused on the end of life (*T*), and disregarded ethical freedom due to a lack of data.

In other words, since we had no data to estimate the values of Eqs 1 and 2 at each time *t*, we estimated Eq 2 at time *T* (i.e., Eq 4), by indirectly testing Eq 1 at time *T* (i.e., Eq 3) in terms of changes of the *R*^2^ value for Eq 4 due to changes in the weight parameters α, β, and γ. Supplementary Materials III in S1 Text provides details of the transformation of Eqs 1 and 2 of the theoretical model into Eqs 3 and 4 of the empirical model. Table 2 provides the linear regression results.

**Table 2:** The empirical estimation of the life model’s regression coefficients.

HEA	Coeff.	Robust Std. Err.	*t*	*P*>|*t|*	[95% Conf. Interval]	
HAP	0.978772	0.1475821	6.63	<0.001	0.6876815	1.269863
FS	0.1059854	0.0969787	1.09	0.276	-0.085295	0.2972659
CS	-0.1645908	0.1243237	-1.32	0.187	-0.4098065	0.0806249
CO	-1.105051	0.3555441	-3.11	0.002	-1.806325	-0.403777
PA	-0.9797172	0.2570708	-3.81	<0.001	-1.486763	-0.4726716
MP	-0.7120002	0.1858725	-3.83	<0.001	-1.078615	-0.345386
BC	-0.8250908	0.3019858	-2.73	0.007	-1.420727	-0.229455
CONS	2.526474	1.261352	2.00	0.047	0.0385869	5.014361

Sample size = 200. Adjusted *R*^2^ = 0.81 (P < 0.01). Abbreviations: CS, creator economic status; CONS, regression intercept; FS, birth family economic status; HAP, happiness; HEA, health. Creator groups: CO = composers of music, PA = painters, MP = mathematicians or physicists, and BC = biologists or chemists.

Note that applying β at 0.5, 0.75, and 1 produced *R*^2^ = 0.61, 0.81, and 0.89, respectively. As expected, the Epicurus component of happiness had the greatest importance for the creators. However, we will refer to the intermediate level (0.75) in our subsequent analysis, since *R*^2^ increases at a decreasing rate for β in [0.5, 1]. In other words, the greatest improvement of the *R*^2^ value occurred between the lowest value (β = 0.5) and the intermediate value of β (β = 0.75), while the value of β that produced the maximum *R*^2^ (β = 1) treats the weights of α and γ as zero. Moreover, the economic status of the creator’s birth family (FS) and the creator’s economic status (CS) had the expected positive and negative impacts (i.e., a richer family, denoted by a larger FS, and a better acceptance of the family status, denoted by a smaller CS minus FS, will increase health). Finally, all creators achieve an expected lower level of health with respect to a representative individual involved in other activities, which is consistent with the assumptions about the negative effects of the artistic and scientific activities under consideration on a creator’s health.

## Results

In the previous section, we obtained consistent data for each creator by empirically estimating a theoretical model based on an original dataset. However, the statistical analysis of the relationship between long-run social benefits (SOC) and individual costs in terms of HEA and HAP requires data on long-run social impacts for each creator. In this section, we will meet this requirement by comparing the relationships estimated by graphical regression analysis and the determinants identified by linear statistical analysis based on a consistent variable across domains for long-run social benefits.

Since the targets of art and science are different (i.e., the general population is the audience for music and painting, the scientific community is the audience for science), we standardized the long-run social impacts within each creator group to allow comparisons between domains. In particular, we calculated SOC for CO as the average number of works performed per year between 2009 and 2019, divided by the number of performances of Beethoven (2975); for PA, SOC was expressed as the number of paintings exhibited in the 10 most popular museums as a percentage of the number of Van Gogh paintings exhibited in the same museums (67); for mathematics, physics, biology, and chemistry, SOC was calculated as the average number of citations between 2009 and 2019 reported in the Scopus database (scopus.com) divided by the number of citations of Poisson (6001), Einstein (2345), Darwin (345), and Gibbs (2407), respectively.

Note that we used citations that occurred in the title, abstract, or keywords of articles (i.e., we excluded reviews), since data are not available for citations of each single publication (i.e., books and articles) published in the 19th century. Moreover, the reference mathematician (Poisson) was cited to a greater extent than other reference scientists (see the Discussion for more details), although the long-run social benefit SOC did *not* increase significantly with increasing birth year (BY) (the slope of the linear regression was –0.005 with *t* at –0.93 and *P* > 0.35), which highlights the creative homogeneity of the period under consideration. Finally, Marie (Curie) Sklodowska belonged to both the MP and BC groups, since she undoubtedly was both a physicist (her Nobel prize in 1911) and a chemist (her Nobel prize in 1903).

### Different individual costs for social benefits in different domains

Let us make three reasonable assumptions (which we will subsequently test) that account for the characteristics of the period under consideration. First, convergent thinking is greater in CO, MP, and BC than in PA. In other words, PA requires a smaller investment in technique, as innovative painters have relied on divergent thinking to a greater extent than on convergent thinking. However, Seurat formally studied optics to guide his painting.

Second, composers have their works performed, painters have their works exhibited, and scientists have their works cited for periods longer than 100 or 200 years (i.e., the selected creators produced long-run social benefits) if they were innovative during their life. Indeed, reviewers for science and critics for art assess the novelty of creators, although with an important difference: reviewers check scientific works before publication, without affecting citations after publication; in contrast, critics affect performances and exhibitions both before and after their first performance and exhibition, although the impacts of critics for music and for painting after the first performance or exhibition of the works are typically for limited contexts (e.g., the period when the creator served as a national composer or painter), limited times (e.g., rediscovery of a forgotten composer or painter, promotion of a composer or painter based on changes in what is seen as fashionable) for some minor creators (i.e., creators with small percentages of performances and exhibitions relative to the numbers for Beethoven and Van Gogh), but never for world-class artists in the long-run (i.e., art critics play only a small role in the long-run).

Third, we assumed that the general population is more important for creators in CO and PA than for creators in MP and BC. That is, composers and painters are creating for the general population, not their peers, and therefor encounter greater communication (language) issues with their audience, whereas scientists communicate primarily with their peers, and therefore have fewer language problems. However, Schoenberg formally studied acoustics to guide his music.

In other words, the main difference between CO and PA, which are characterized by similar levels of language issues when communicating with non-colleagues, is the relatively larger importance of convergent thinking in CO and divergent thinking in PA, whereas the main difference between CO and scientists, characterized by the same relative importance of convergent thinking, is the more important language issues communicating with non-colleagues for CO than for communicating with peers for scientists.

Some remarks on the role of aesthetics are worth considering here to support these three assumptions. Although music and painting do not follow a set of codified and consistent rules to communicate their meaning (i.e., they are emotional rather than semantic), they do use a set of codified and consistent objects (e.g., dominant or diminutive seventh chords, major and minor tonalities, warm and cold colors, perspective or plain drawing) *to intentionally or unintentionally evoke emotions*, *feelings or affects* in their audience of listeners or viewers [23, 24]: these objects depend on the society in which they are produced (i.e., the sedimentation of objects for composers and painters, the appreciation of works based on these objects for the general population).

Note that the nature of art is irrelevant for the purposes of the present study, since we focus on social benefits in terms of performances and exhibitions and the associated health and happiness costs for the individual creator. Moreover, any referential language has some evocative effect, apart from unequivocal technical definitions (e.g., “partial derivatives” has only one meaning in mathematics, whereas “sea” evokes different meanings according to the individual’s different experience and knowledge). Finally, the knowledge gained from art is marginal for the present study, since “emotion” could be replaced with “understanding” without affecting our analysis.

In particular, artistic creations are intentionally or unintentionally related to emotional experiences (i.e., artists who compose or paint for themselves do not consider the emotions evoked in others), although emotional connections are insufficient to explain the expressivity of music and painting, which represents the capacity of artistic works to provoke emotions; that is, there is no aesthetics of expression in philosophy (e.g., [25]), although there might be an exhaustive computer description of emotional factors in informatics (e.g., [26]). This is because emotions are based on synesthesia (i.e., interrelationships between senses that differ among individuals) and because listeners hear what they have learned to hear and viewers see what they have learned to see; that is, music and painting do not say something separate from themselves but rather express something inside themselves, and they require individual interpretation, which is closer to a mimetic operation rather than to an analytic operation. Thus, the expressivity of music and painting (i.e., the capacity of artistic works to provoke emotions in the general population) changes over time (due to advances in artistic techniques and education of viewers and listeners) and changes over space due to differences across and within cultures. For example, there have been periods of popular appraisal by many members of society rather than only its elite members, as in the case of the 5 days of the Milan insurrection in 1848, when young people sang contemporary operas by Verdi. There have also been periods of popular appraisal when primarily the elite members of society appreciated the art, as in the case of young people today, who sing neither the past operas by Verdi nor contemporary symphonies by Busoni. However, advances in artistic techniques intermittently provide pressure to improve education, as in the cases of the dodecaphonic music by Schoenberg, which promoted the appraisal of diatonic music by Ravel.

These language issues do not exist in the sciences, where the language rules have been codified and are shared by all scientists. For example, the language of mathematics is standardized and used by all scientists to communicate clearly.

However, composers whose works are still performed and painters whose works are still exhibited after 200 years were clearly “authentic and autonomous” in Adorno’s [27] words; that is, they were alienated by their creativity rather than being inauthentic and heteronomous. Here, we have used *authentic* to mean original rather than derivative of previous works. In other words, they use an *original* language and provoke *original* emotions. Thus, the study of the grammar and syntax of music and painting is not only about Western art. For example, the compositions of De Sarasate, who used a language close to that of Saint-Saens and Lalo (i.e., with similar evoked emotional experiences), are performed less often than those of the latter two composers, since Saint-Saens and Lalo introduced that music language before De Sarasate, as has been highlighted by critics. Similarly, Serusier used a language close to that of Gauguin and Van Gogh (i.e., with similar evoked emotional experiences), but is exhibited to a smaller extent, since Gauguin and Van Gogh introduced that visual language before Serusier, as has been highlighted by critics.

In particular, since *the number of potential innovative techniques* that can be used to introduce an original language and provoke original emotions is smaller for painters than for composers, painters who want to be innovative rely to a relatively greater extent on divergent thinking (e.g., Mondrian) and to a relatively smaller extent on convergent thinking (e.g., Turner), whereas composers rely to a relatively greater extent on convergent thinking (e.g., Schubert) and to a relatively smaller relative extent on divergent thinking (e.g., Beethoven).

Therefore, the creator’s contemporaries might have encountered difficulty feeling the emotions intended by authentic artists, and as a result, these authentic artists did not achieve economic, professional, or social success (i.e., they would have smaller HAP and HEA). Conversely, the creator’s contemporaries might have no problems in feeling the emotions intended by inauthentic artists, and inauthentic artists might therefore have achieved economic, professional, or social success (i.e., larger HAP and HEA).

**Fig 1:**
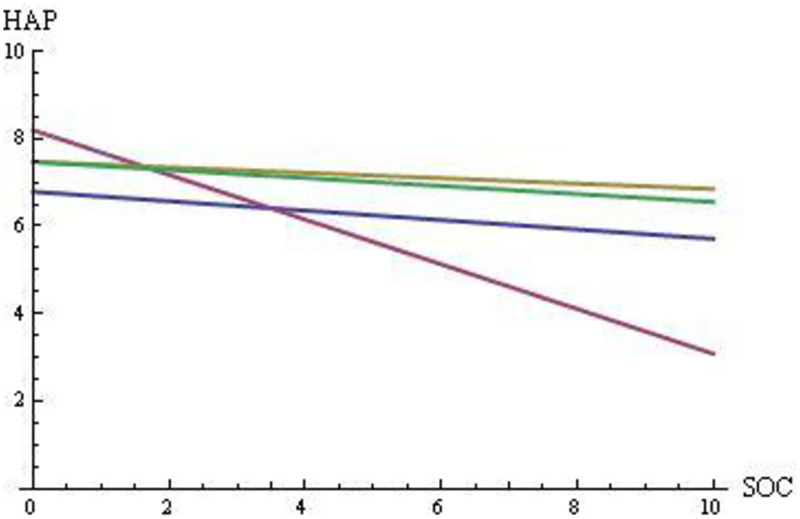
Social benefits (SOC) in [0, 10] vs. happiness (HAP) in [0, 10] if the relative contributions to happiness have values of α = 0.125, β = 0.75, and γ = 0.125. For composers (blue) CO: HAP = -0.107 SOC + 6.785; for painters (purple) PA: HAP = -0.511 SOC + 8.20; for mathematicians and physicists (yellow) MP: HAP = -0.062 SOC + 7.467; and for biologists and chemists (green) BC: HAP = -0.091 SOC + 7.466.

**Fig 2:**
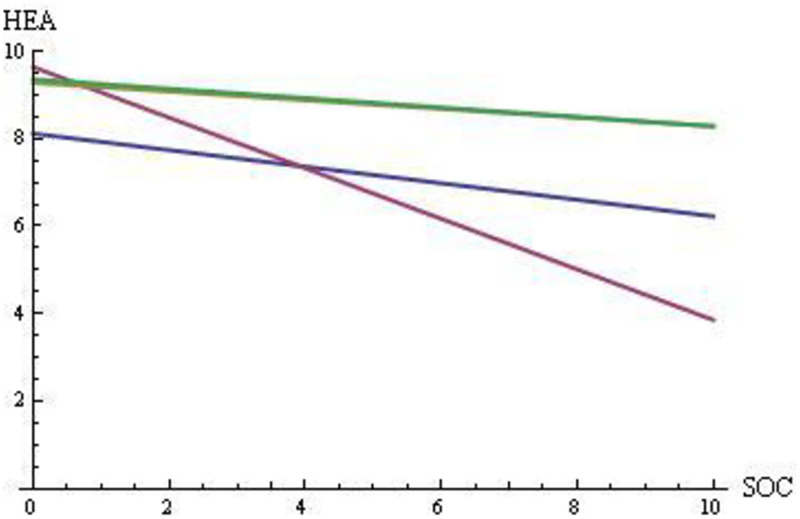
Social benefits (SOC) in [0, 10] vs. health (HEA) in [0, 10] if the relative contributions to happiness have values of α = 0.125, β = 0.75, and γ = 0.125. For composers (blue) CO: HEA = -0.189 SOC + 8.115; for painters (purple) PA: HEA = -0.578 SOC + 9.633; for mathematicians and physicists (yellow) MP: HEA = -0.099 SOC + 9.2763; and for biologists and chemists (green) BC: HEA = -0.107 SOC + 9.355.

In summary, painters must rely to a greater extent on divergent thinking (which has a larger impact on HEA and HAP) and painters encounter language issues (that decrease HAP and HEA); composers can rely to a greater extent on convergent thinking (which has a smaller impact on HEA and HAP) even though composers also face language issues; and scientists can rely to a greater extent on convergent thinking (which has smaller impact on HEA and HAP) and face few language issues. Based on the analysis described above, we defined the relationships between happiness, health, and social benefit for the four groups of creators. Fig 1 shows the relationship between HAP and SOC, and Fig 2 shows the relationship between HEA and SOC. Supplementary Materials V in S1 Text (S1–S8 Figs in S1 Text) provides the graphical regression equations used to generate these lines.

Note that we have presented linear relationships, which is consistent with the linear theoretical models developed in the Materials and Methods: these lines include the effects of different institutions (e.g., employment status) for different groups of creators.

**Fig 3:**
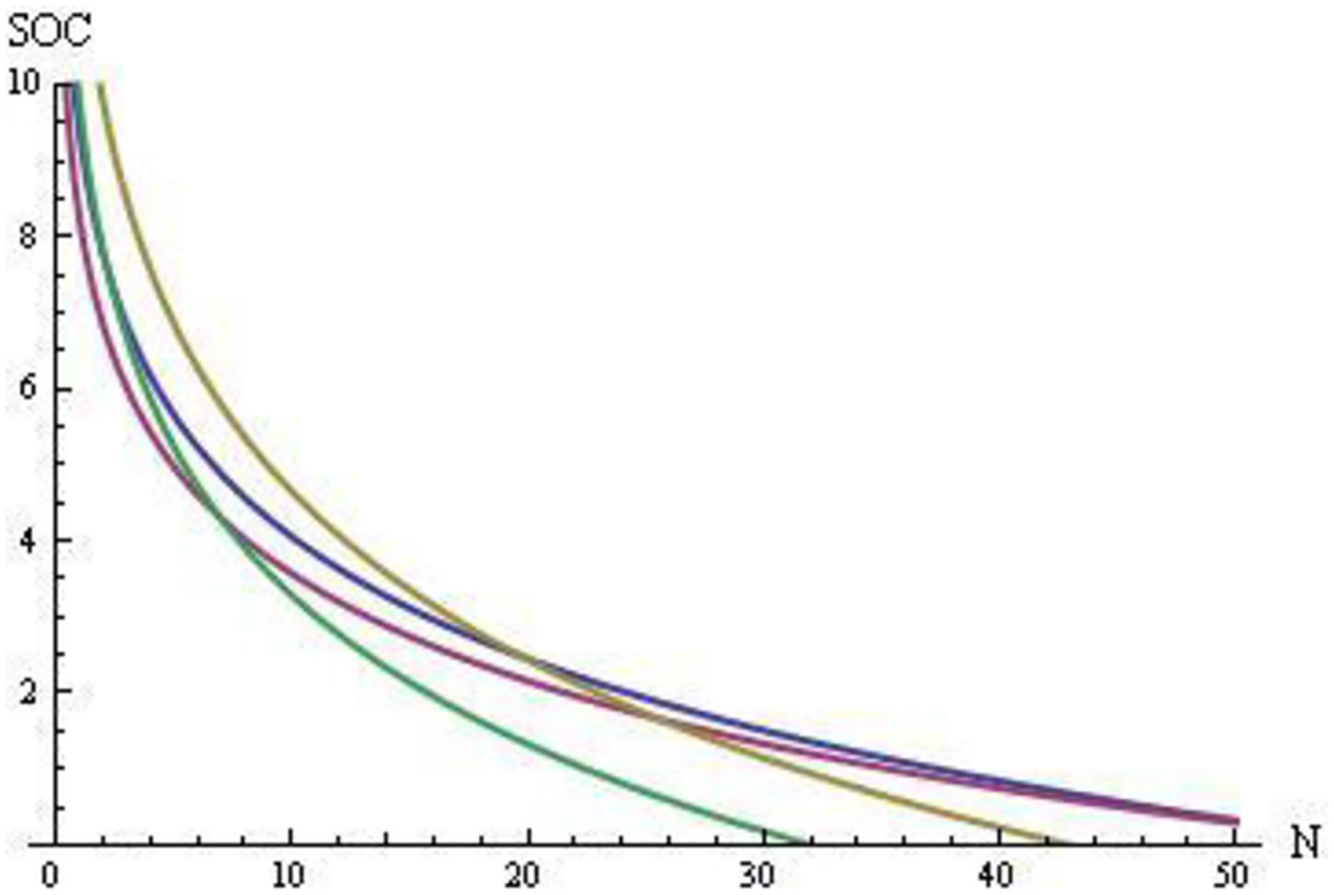
Social benefits (SOC) in [0, 10] vs. the number of creators in each group (N) in [0, 50]. For composers (blue) CO: SOC = -2.319 ln[N] + 9.359; for painters (purple) PA: SOC = -2.038 ln[N] + 8.263; for mathematicians and physicists (yellow) MP: SOC = -3.193 ln[N] + 12.005; and for biologists and chemists (green) BC: SOC = -2.826 ln[N] + 9.792. S9–S12 Figs in S1 Text provides the graphical regression equations for the relationships between SOC and N in the four domains.

Several insights can be obtained by comparing the regression lines for the four domains in terms of their intercepts and slopes:

- Creativity (and social benefits SOC) has a cost to the individual creator in terms of HEA and HAP in all domains (i.e., all slopes are negative).
- The HAP line is lower (farther from the maximum value of 10) than the HEA line for all creator groups; indeed, the health status (HS) is an original beneficial stock for HEA (i.e., HS is an essential component of HEA, as it represents the starting value for HEA), whereas HAP does not benefit from a similar initial stock.
- The PA lines are above the other lines for HAP and HEA at a low level of creativity (and of social benefits SOC); thus, painting is good for health and happiness, with language issues playing a smaller role for painters who are more conventional and less creative.
- For both HEA and HAP, painters bear the largest cost of creativity (PA shows the largest negative slope); this may be because painters rely to the greatest extent on divergent thinking.
- The CO lines are below the MP and BC lines; indeed, composers face larger language issues than scientists since they refer to the general population rather than to their colleagues (i.e., larger costs to the individual at each level of creativity). The larger values of psychological and somatic problems (i.e., 31, 20, 7, and 7 for CO, PA, MP, and BC, respectively, in Table 1) support this insight.
- The CO lines are slightly steeper than the MP and BC lines, suggesting a greater cost to HAP and HEA with increasing SOC: indeed, convergent thinking is more important than divergent thinking in these domains, but language issues in CO are slightly smaller for less creative and more conventional composers.
- MP had a slightly higher HAP than BC; indeed, the creations of mathematicians and physicists are cited for longer durations, on average (see Fig 3) (i.e., in Fig 1, larger SOC at the same level of HAP).
- MP shows the same level of HEA as BC: indeed, mathematicians and physicists are cited longer, but biologists and chemists had a better health status (i.e., maximum HS at 80% and 86% for MP and BC, respectively, in Table 1; similarly, mean HS at 2.78 and 2.86 for MP and BC, respectively, in Table 1).
- The PA line is below the CO line (although PA and CO face similar language problems) and the PA line is below the MP and BC lines (although MP and BC are not affected by language problems) at a high level of creativity (and of social benefits SOC); indeed, a larger number of PA than other creator groups had HS = 2 and PP = 1 (i.e., in Table 1, Chopin and Mendelssohn for CO; Friedrich, Toulouse-Lautrec, Troyon, and Van Gogh for PA; and Cantor for MP).

These results, combined with our three assumptions, lead to the following three insights. First, if creativity in PA is based to a greater extent on divergent thinking than on convergent thinking, since the PA line has the largest slope as a function of SOC, *then* creativity from divergent thinking is more demanding in terms of HEA and HAP. In other words, *creativity based on divergent thinking increases the steepness*. However, this may be because painters also face language issues. Second, if creativity in CO is based to a greater extent on convergent thinking, as it is in science, even though CO is characterized by larger language issues than MP and BC, since the CO line has a similar slope to the MP and BC lines, but has a smaller intercept, *then* creativity from convergent thinking has a lower cost in terms of HEA and HAP, although language issues effect both HEA and HAP. In other words, *language issues decrease the intercept*. Third, combining the two previous insights with Figs 1 and 2 at high levels of SOC and at low levels of both HEA and HAP suggest that *long-run social benefits arise to a greater extent from creativity based on divergent thinking*, regardless of any language issues that characterize the different creator groups.

### Similar individual costs for *net* social benefits in different domains

In the previous sub-section, we discussed the costs of creativity to the individual for the four domains. However, Table 1 showed different characteristics for the four creator groups, with a single variable representing a social cost (i.e., the employment status EM). In this sub-section, we will test whether the relationships between SOC and the group’s HEA or HAP become similar if they are separated from the impacts of the employment status (EM), and will also consider the effect of the marital status (MA). Note that all variables other than SOC, HEA, and HAP are dummy variables in [0,1]. We chose this approach to increase variability, since the sample size is small (i.e., 200 observations). Tables 3 and 4 provide linear regression results of the following equations:

$$\text{HAP}=\text{CONS}+\text{SOC}+\text{EM}+\text{MA}+\text{CO}+\text{PA}+\text{MP}+\text{BC}+\zeta_{\text{HAP}}$$ (5)

$$\text{HEA}=\text{CONS}+\text{SOC}+\text{EM}+\text{MA}+\text{CO}+\text{PA}+\text{MP}+\text{BC}+\zeta_{\text{HEA}}$$ (6)

where ζ_HAP_ and ζ_HEA_ represent stochastic error terms.

**Table 3:** The empirical estimation of the individual happiness (HAP) costs for social benefits (SOC) using Eq 5.

HAP	Coeff.	Robust Std. Err.	*t*	*P*>|*t|*	[95% Conf. Interval]	
SOC	-0.1278457	0.0453425	-2.82	0.005	-0.217279	-0.0384123
EM	0.7351393	0.3010054	2.44	0.016	0.1414373	1.328841
MA	0.1018215	0.2480316	0.41	0.682	-0.3873953	0.5910382
CO	-2.465993	0.4306435	-5.73	<0.001	-3.315392	-1.616593
PA	-1.610017	0.4423877	-3.64	<0.001	-2.482581	-0.7374536
MP	-1.734621	0.2892077	-6.00	<0.001	-2.305053	-1.164189
BC	-1.447709	0.2277914	-6.36	<0.001	-1.897004	-0.9984138
CONS	8.751568	0.4073099	21.49	<0.001	7.948192	9.554945

Sample size = 200. Adjusted *R*^2^ = 0.13 (P < 0.01). Robust Standard Errors = Huber/White estimators. Abbreviations: EM, employment status; MA, marital status, CONS, regression intercept. Creator groups: CO = composers of music, PA = painters, MP = mathematicians or physicists, and BC = biologists or chemists.

**Table 4:** The empirical estimation of the individual health (HEA) costs for social benefits (SOC) using Eq 6.

HEA	Coeff.	Robust Std. Err.	*t*	*P*>|*t|*	[95% Conf. Interval]	
SOC	-0.1652824	0.0524441	-3.15	0.002	-0.268723	-0.0618418
EM	0.5044576	0.350251	1.44	0.151	-0.1863763	1.195291
MA	-0.1486146	0.2801023	-0.53	0.596	-0.7010874	0.4038582
CO	-3.521048	0.4571447	-7.70	<0.001	-4.422719	-2.619377
PA	-2.660581	0.4951863	-5.37	<0.001	-3.637285	-1.683877
MP	-2.315452	0.2395622	-9.67	<0.001	-2.787963	-1.84294
BC	-2.223893	0.2816868	-7.89	<0.001	-2.779491	-1.668295
CONS	11.33853	0.4636383	24.46	<0.001	10.42405	12.25301

Sample size = 200. Adjusted *R*^2^ = 0.15 (P < 0.01). Robust Standard Errors = Huber/White estimators. Abbreviations: EM, employment status; MA, marital status, CONS, regression intercept. Creator groups: CO = composers of music, PA = painters, MP = mathematicians or physicists, and BC = biologists or chemists.

Thus, controlling for other factors, the long-lasting social impact SOC measured by our indexes (the relative numbers of performances, exhibitions, and citations) is associated with decreased individual happiness HAP and health HEA (as measured by our indexes of happiness and health), and the impacts were negative and statistically significant for all creator groups, although each creator group was characterized by a different impact size.

Note that the employment status (EM) has a positive impact for both HAP and HEA (i.e., it is a social cost that compensates for individual costs in terms of happiness and health), and although this was significant for HAP (*P* = 0.016), it was not significant for HEA (*P* = 0.151). The employment status (EM) can determine whether a creator has a lower potential loss from embracing a life of exploration because they have a supplemental income that lets them be creative without having to worry about whether they can earn enough money to survive (e.g., employment at a conservatory for CO, an academy for PA, or a university for MP and BC). The marital status (MA) had no significant impact on both HEA and HAP. We confirmed the robustness of our results by using the birth year (BY) as a control variable. Indeed, BY did not significantly affect HEA or HAP in Eqs 5 and 6, and all significant variables included in Eqs 5 and 6 were the same whether or not we included BY, with the impacts on HEA and HAP of the four domains in the same order with or without BY, while the regression intercept was significant in the estimations without BY but not in the estimations with BY. Supplementary Materials IV in S1 Text provide a sensitivity analysis that supports these conclusions.

Thus, the slope of the regression for PA is between those of BC and MP in terms of HEA and between those of MP and CO in terms of HEA. In other words, it is not possible to distinguish science from art in terms of their HAP and HEA costs.

## Discussion

Many insights were provided by our study:

- Creativity is a cost to the individual in all domains, but has a larger impact on HAP than on HEA.
- The cost is larger if creativity is based more on divergent thinking than on convergent thinking.
- The cost is larger if creators face greater language issues when they attempt to communicate with their audience.
- Psychological problems do not depend on the success lag (SY–BY; the Pearson’s correlation *r* between this variable and PP was *r* = 0.11).
- The duration of social benefits was larger for the two artistic groups than for the two scientific groups, as expected, and the number of creators with an important SOC was ranked as CO ≅ PA > MP > BC (Fig 3) based on the graphical regression equations presented in Supplementary Materials V in S1 Text. Indeed, scientists are no longer cited after their suggested methodologies or their obtained breakthroughs become common practice or common knowledge. For example, the graphical framework (orthogonal *x* and *y* axes) developed by Descartes is no longer cited as his work in publications that rely on this framework.
- Long-run social benefits do not depend on life years (DY–BY; the Pearson’s correlation *r* between this variable and SOC was *r* = –0.07).

Note that there are both differences and similarities between the present study and the literature on exploration, which refers to search, variation, risk taking, experimentation, flexibility, discovery, and innovation, and the literature on exploitation, which refers to refinement, choice, production, efficiency, selection, implementation, and execution [28]. In terms of the differences, we lacked detailed information about the sequence of creation characteristics for composers and painters and the sequence of research topics for scientists that would let us test for a possible sequence of exploration followed by exploitation [29]. In addition, we focused on long-run social benefits rather than on short-run individual careers [29]. In terms of similarities, we could link exploration with divergent thinking and exploitation with convergent thinking [30], and rephrase our *positive* results (i.e., explanatory insights) as long-run social benefits arise to a greater extent from exploration than from exploitation (i.e., the social benefits that arise from search and innovation are greater than those that arise from refinement and efficiency), although creativity is based on both divergent and convergent thinking. In addition, we could link risk taking with divergent thinking [30], and rephrase our *normative* results (i.e., advisory insights) as society should bear the risk of a creator’s explorative life by providing guaranteed employment (i.e., incentives should be offered to creators who take on personal risks to produce results with social benefits), even though creative individuals appear to be less risk averse than the average individual.

The main weaknesses of our study are:

1. It is based on a *specific* period. However, a period before the chosen period (i.e., 1770–1879) would exclude all painters and many composers, since they would refer primarily to creators who have a single patron; that is, artistic works were thought to *ex ante* satisfy most of the requirements of artistic demand, apart from some details deliberately introduced by the artists, as in the case of Michelangelo’s Sistine Chapel. This contrasts with our (third) assumption that the general population is more important for creators in CO and PA than for creators in MP and BC, with creators in MP and BC having fewer language problems than creators in CO and PA; that is, artistic works were mostly driven by artistic insights or inspirations, which could *ex post* meet the expectations of the artistic demand to a greater or smaller extent, as in the case of Bizet’s opera “Carmen”. Our motivation for choosing this period was to include Beethoven and Einstein as the reference creators for music and physics, respectively; choosing a longer period to increase the balanced sample would require the addition of less-popular museums, since more recent painters are not well-represented in the 10 most popular museums.
2. The arts and sciences that we analyzed had attained different degrees of maturity during the period under consideration. Both music and painting are depicted from adolescence to maturity (i.e., from Beethoven to Schoenberg and from Turner to Klee) if we define music as “the art of combining vocal and/or instrumental sounds to produce beauty of form, harmony, and expression of emotion” (Oxford English Dictionary) before Ligeti (when performers could choose what to play within a specified range of sounds for a specified period of time), and if we define painting as “the practice of applying paint, pigment, color, or another medium to a solid surface” (Cambridge English Dictionary) before Fontana (when a two-dimensional surface was replaced by a three-dimensional solid). In contrast, the sciences are depicted from their infancy (e.g., biology and chemistry) to their adolesence (e.g., mathematics and physics). However, we considered social benefits *today* after 200 to 100 years have passed, which is a sufficiently long period to allow a comparison of these different creator groups.
3. The role of critics for painting and music (as gate-keepers and fame-constructors) and the role of musicians for music (as interpretation-prompters) are only considered *implicitly*. The social benefits could be indirectly estimated by the willingness to pay for concert or museum tickets or, in other words, the willingness to pay for the suggested interpretation of music in concerts performed by musicians and for the suggested interpretation of paintings in exhibitions organized by critics (i.e., the estimated social benefits are conditional to critics and musicians). However, philological studies in both music and painting are likely to lead to similar interpretations of art in the long-run, with critics and musicians unable to affect the social benefits of art in the long-run. For example, many composers and painters who had been dismissed by contemporary critics were rediscovered after their death, whereas musicians with unusual performances were often ignored by subsequent generations of musicians.

Note that we could not rely on direct observation of a creator’s happiness during their life, so we measured HAP indirectly by referring to economic, professional, and social achievements. However, possible dissatisfaction with these achievements could be depicted by the health component of happiness.

The main strengths of the present study are:

1. Our approach let us compare very different groups of creators by accounting for very different audiences (i.e., the scientific community for MP and BC; the general population for CO and PA).
2. The analysis reveals the long-run impacts of creativity on social benefits instead of using careers to judge individual benefits in the short-run.
3. The consideration of both HEA and HAP as individual costs incurred to obtain social benefits reveals tight links with convergent thinking (prevalent in CO, MP, and BC) and divergent thinking (prevalent in PA).

In addition, our observation that the employment status (EM) functions as a statistically significant social cost for HAP (and HEA, to a lower extent) suggests that policies could be developed to reduce institutional differences among the four creator groups. That is, creators who are funded and who do not need to work outside their field to earn their living can improve social benefits at a lower cost to their happiness (and to their health, to a lower extent).

## Conclusions

The main overall insight we obtained is that there is a significant statistical impact of the long-run social benefits from creativity on an individual creator’s costs in terms of health and happiness, and that this cost does not differ greatly between the arts and the sciences if institutional differences are taken into account. In particular, painting was closer to science than it was to music in terms of its personal costs. We explained this feature by stressing that composers face language issues when communicating with non-colleagues (i.e., the general population), that composing is not good for the creator’s health (i.e., it requires a large investment in technique for even a low level of creativity), and that composers are employed at a conservatory or academy to a smaller extent than scientists are employed at a university. In other words, the difference among groups of creators depends on the institutional context rather than on the creative process; that is, employment at a conservatory, academy, or university could reduce these differences.

Moreover, creators in all four groups exhibit heroic behavior in the sense that they bear individual costs to their happiness and health to provide social benefits for the rest of society, although they are likely to be driven by an urge to explore or a desire for immortality.

Finally, there has been continuous support for all four groups of creators by the general population and by expert colleagues across domains, although the former prevails in art and the latter in science. Consider Beethoven, who is both cited by other composers in their compositions and famous to the general population, and consider Einstein, who is both cited by other scientists in their articles and famous to the general population. Since happiness depends on short-run economic achievements (the Aristotle component), professional achievements (the Epicurus component), and social achievements (the Zeno component), and since health decreases if creativity is based to a greater extent on divergent thinking than on convergent thinking, the high levels of both health and happiness at a low level of long-run social benefits suggest that both the creator’s *contemporary* general population for art and the *mainstream* scientific community for science appreciate creativity arising from convergent thinking to a greater extent than creativity arising from divergent thinking. For example, consider the wandering harmony in Beethoven’s work, and general relativity in Einstein’s work.

## Supporting information

S1 Text Benefits and costs of creativity. (DOCX)

S1 Data (TXT)
