# Supplementary material for: Social benefits and individual costs of creativity in art and science: A statistical analysis based on a theoretical framework
Source: PLoS One. 2022 Apr 27;17(4):e0265446. doi: 10.1371/journal.pone.0265446 (PMC9045641; doi:10.1371/journal.pone.0265446)
Supplement: S1 Text — (DOCX) [file pone.0265446.s001.docx]

# Supplementary Materials I: The list of empirical variables

Names, meanings and units of variables.

| Name | Meaning | Units or range |
| --- | --- | --- |
| α | Aristotle component of happiness | [0,1] |
| β | Epicurus component of happiness | [0,1] |
| γ | Zeno component of happiness | [0,1] |
| α* | The assumed average empirical value of α | 0.125 |
| β* | The assumed average empirical value of β | 0.75 |
| γ* | The assumed average empirical value of γ | 0.125 |
| ε | The stochastic error term in equations 4 | HEA |
| ζ_HAP,_ ζ_HEA_ | Stochastic error terms in equations 5 and 6 | HAP, HEA |
| AY | Year of the first social achievement | year |
| BC | Biologists and chemists | 0 or 1 |
| BY | Birth year | year |
| CO | Composers of music | 0 or 1 |
| CS | Economic status of the creator’s family | 1 to 3 |
| DY | Death year | year |
| EM | Employment status | 0 or 1 |
| FS | Economic status of the original family | 1 to3 |
| HAP | Happiness level | 0 to10 |
| HEA | Health level | 0 to10 |
| HS | Health status | 1 to 3 |
| LY | Life duration | year |
| MA | Marital status | 0 or 1 |
| MP | Mathematicians and physicists | 0 or 1 |
| OC | Occupation type | CO, PA, MP, BC |
| PA | Painters | 0 or 1 |
| PP | Psychological problems | 0 or 1 |
| SOC | Long-run social benefits | 0 to10 |
| SP | Somatic problems | 0 or 1 |
| SY | Year of the first professional achievement | year |
| *T* | End of life year | year |

# Supplementary Materials II: The dataset of artists and scientists

Detailed data are available from the authors on request for the following authors:

Composers (*N* = 50): Albeniz, Beethoven, Bellini, Berlioz, Bizet, Borodin, Brahms, Bruch, Bruckner, Busoni, Cajkovskij, Chopin, De Sarasate, Debussy, Donizetti, Dvorak, Elgar, Fauré, Frank, Grieg, Lalo, Leoncavallo, Lizt, Mahler, Mascagni, Massenet, Mendelssohn, Mussorgsky, Offenbach, Paganini, Ponchielli, Puccini, Rachmaninoff, Ravel, Reger, Respighi, Rimskij-Korsakov, Rossini, Saint-Saens, Satie, Schoenberg, Schubert, Schumann, Smetana, Spohr, Strauss (Johann), Strauss (Richard), Verdi, Von Weber, Wagner.

Painters (*N* = 50): Anquetin, Bazille, Bernard, Bingham, Bocklin, Bonnard, Burne-Jones, Cézanne, Church, Constable, Corot, Courbet, Degas, Delacroix, Denis, Derain, Flandrin, Friedrich, Gauguin, Géricault, Guerin, Homer, Huet, Ingres, Kandinsky, Klee, Klimt, Lacombe, Manet, Matisse, Millet, Mondrian, Monet, Moreau, Pissarro, Ranson, Renoir, Rousseau, Roussel, Sargent, Serusier, Seurat, Signac, Sisley, Toulouse-Lautrec, Troyon, Turner, Van Gogh, Vuillard, Whistler.

Mathematicians (*N* = 26): Abel, Bolzano, Boole, Cantor, Cauchy, Cayley, Dirichlet, Frege, Frobenius, Galois, Gauss, Hamilton, Hermite, Klein, Landau, Laurent, Mobius, Peano, Poincaré, Poisson, Riemann, Stokes, Volterra, Van Der Waals, Weierstrass, Zermelo.

Physicists (*N* = 24): Boltzmann, Bragg, Braun, Carnot, Clausius, Curie, Einstein, Faraday, Goldstein, Guillaume, Hertz, Joule, Kelvin, Kirchhoff, Maxwell, Millikan, Planck, Rayleigh, Röntgen, Rutherford, Sklodowska, Stark, Thomson, Zeeman.

Chemists (*N* = 36): Arrhenius, Avogadro, Baekeland, Berzelius, Bosch, Buchner, Bunsen, Cannizzaro, Davy, Fisher, Frankland, Gibbs, Grignard, Haber, Hess, Kekulé, Kolbe, Loschmidt, Mendeleev, Nernst, Nobel, Ostwald, Parkes, Perkin, Prout, Ramsay, Richards, Sabatier, Sklodowska, Soddy, Solvay, Sorensen, Van 't Hoff, Wage, Wallach, Werner.

Biologists (*N* = 15): Bernard, Cvet, Darwin, De Morgan, De Vries, Flemming, Koch, Liebig, Mendel, Pasteur, Schleiden, Virchow, Von Tschermak, Weismann, Wohler.

# Supplementary Materials III: From theoretical to empirical models

We transformed the theoretical model (i.e., equations 1 and 2) into an empirical model (i.e., equations 3 and 4) as follows. Equation 1 of the theoretical model is transformed into equation 3 in the empirical model as follows: In addition to assuming that HEA equaled 75% of HAP (as detailed in the main text), *y*[*T*] = CS (i.e., the creator’s income is measured by their economic status); (*y*[*t*] – *y*[*t*–1]) / *y*[*t*–1] becomes (DY – SY) / LY (i.e., the creator’s absolute achievement is measured by the successful period of their life, expressed as a percentage); (*y*[*t*] – *ay*) / *ay* becomes (DY – AY) / LY (i.e., the relative creators’ achievement is measures by the awarded period of their life, expressed as a percentage). Moreover, equation 2 of the theoretical model is transformed into equation 4 in the empirical model as follows: In addition to estimating HAP at final *T* (as detailed in the main text), we disregarded *os* due to a lack of data on the individual set of opportunities; we disregarded δ (*y*[*t*] – *y*[*t* – 1]) due to a lack of data on the psychological stress caused by missed achievements; *ed* becomes FS (i.e., education is approximated by the original family’s socioeconomic status); *y*[*T*] = CS (i.e., the creator’s income is measured by their economic status); and *oc* = OC (i.e., occupation depicts the four creative domains). Finally, we disregarded uncertainty because a creator’s HAP and HEA are estimated backwards from their death year.

# Supplementary Materials IV: Sensitivity analyses

We tested whether the employment status (EM) affected the overall significance of the life model obtained in the Materials and Methods. For regressions that included and excluded EM, *R*^2^ was the same (Table S1).

Table S1. The empirical estimation of the life model coefficients for an analysis that included employment status (EM). Sample size = 200. Adjusted *R*^2^ = 0.81. Abbreviations: CS, creator economic status; CONS, regression intercept; FS, birth family economic status; HAP, happiness; HEA, health. Creator groups: CO = composers of music, PA = painters, MP = mathematicians or physicists, and BC = biologists or chemists.

| HEA | Coeff. | Robust Std. Err. | *t* | *P*>*t* | [95% Conf. Interval] | |
| --- | --- | --- | --- | --- | --- | --- |
| HAP | 0.9899262 | 0.1527861 | 6.48 | <0.001 | 0.6885615 | 1.291291 |
| FS | 0.1029339 | 0.0965951 | 1.07 | 0.288 | -0.0875964 | 0.2934641 |
| CS | -0.198796 | 0.1307782 | -1.52 | 0.130 | -0.456751 | 0.0591591 |
| EM | -0.2466738 | 0.1424739 | -1.73 | 0.085 | -0.5276981 | 0.0343506 |
| CO | -1.083974 | 0.3611764 | -3.00 | 0.003 | -1.79638 | -0.371567 |
| PA | -1.082157 | 0.2412758 | -4.49 | <0.001 | -1.558065 | -0.6062497 |
| MP | -0.6566457 | 0.2007843 | -3.27 | 0.001 | -1.052685 | -0.2606062 |
| BC | -0.7735149 | 0.3187681 | -2.43 | 0.016 | -1.402273 | -0.144757 |
| CONS | 2.670437 | 1.232004 | 2.17 | 0.031 | 0.2403568 | 5.100517 |

In particular, when we included EM, the four creator types, together with the employment status and the regression intercept, contributed significantly to health.

We also tested whether the birth year (BY) affected the significance of the variables used as determinants of the individual happiness and health obtained in the Results. For regressions that included and excluded BY, *R*^2^ are the same (Tables S2 and S3).

Table S2. The empirical estimation of the individual happiness (HAP) costs for social benefits (SOC) for an analysis that included birth year (BY). Sample size = 200. Adjusted *R*^2^ = 0.13 (P < 0.01). Robust Standard Errors = Huber/White estimators. Abbreviations: EM, employment status; MA, marital status, CONS, regression intercept. Creator groups: CO = composers of music, PA = painters, MP = mathematicians or physicists, BC = biologists or chemists, and BY = the birth year.

| HAP | Coef. | Robust Std. Err. | *t* | *P>\|t\|* | [95% Conf. Interval] | |
| --- | --- | --- | --- | --- | --- | --- |
| SOC | -.12432 | .045823 | -2.71 | 0.007 | -.2147041 | -.0339358 |
| EM | .726413 | .3035393 | 2.39 | 0.018 | .1276932 | 1.325133 |
| MA | .069198 | .2495175 | 0.28 | 0.782 | -.4229658 | .5613618 |
| CO | -2.593001 | .468093 | -5.54 | 0.000 | -3.516297 | -1.669705 |
| PA | -1.757774 | .50168 | -3.50 | 0.001 | -2.747319 | -.7682293 |
| MP | -1.859669 | .3462386 | -5.37 | 0.000 | -2.542612 | -1.176727 |
| BC | -1.579349 | .2683164 | -5.89 | 0.000 | -2.108593 | -1.050105 |
| BY | .0042608 | .004372 | 0.97 | 0.331 | -.0043627 | .0128844 |
| CONS | 1.084214 | 7.801011 | 0.14 | 0.890 | -14.30298 | 16.47141 |

Table S3. The empirical estimation of the individual health (HEA) costs for social benefits (SOC). Sample size = 200. Adjusted *R*^2^ = 0.15 (P < 0.01). Robust Standard Errors = Huber/White estimators. Abbreviations: EM, employment status; MA, marital status, CONS, regression intercept. Creator groups: CO = composers of music, PA = painters, MP = mathematicians or physicists, BC = biologists or chemists, and BY = the birth year.

| HEA | Coef. | Robust Std. Err. | *t* | *P>\|t\|* | [95% Conf. Interval] | |
| --- | --- | --- | --- | --- | --- | --- |
| SOC | -.1633538 | .0529394 | -3.09 | 0.002 | -.2677748 | -.0589329 |
| EM | .4996844 | .3519116 | 1.42 | 0.157 | -.1944479 | 1.193817 |
| MA | -.1664596 | .287151 | -0.58 | 0.563 | -.7328539 | .3999348 |
| CO | -3.590521 | .4776201 | -7.52 | 0.000 | -4.532609 | -2.648434 |
| PA | -2.741403 | .5338208 | -5.14 | 0.000 | -3.794345 | -1.688462 |
| MP | -2.383853 | .2740316 | -8.70 | 0.000 | -2.92437 | -1.843336 |
| BC | -2.2959 | .3186888 | -7.20 | 0.000 | -2.924501 | -1.667298 |
| BY | .0023307 | .0048328 | 0.48 | 0.630 | -.0072018 | .0118631 |
| CONS | 7.144519 | 8.69768 | 0.82 | 0.412 | -10.01132 | 24.30036 |

In particular, when we included BY, the signs of the coefficients and their statistical significance were not affected. BY was not statistically significant and the regression intercept was not statistically significant.

# Supplementary Materials V: Graphical regression results

In the following graphs, α represents the Aristotle weight, β represents the Epicurus weight, and γ represents the Zeno weight, as described in the Materials and Methods.

**Figure S1. Social benefits (SOC) in [0, 10] vs. happiness (HAP) in [0, 10] for music composers if the weights are α = 0.125, β = 0.75, and γ = 0.125: HAP = -0.1075 SOC + 6.7848.**

**Figure S2. Social benefits (SOC) in [0, 10] vs. health (HEA) in [0, 10] for music composers if the weights are α = 0.125, β = 0.75, and γ = 0.125: HEA = -0.189 SOC + 8.1149.**

**Figure S3. Social benefits (SOC) in [0, 10] vs. happiness (HAP) in [0, 10] for painters if the weights are α = 0.125, β = 0.75, and γ = 0.125: HAP = -0.5108 SOC + 8.1921.**

**Figure S4. Social benefits (SOC) in [0, 10] vs. health (HEA) in [0, 10] for painters if the weights are α = 0.125, β = 0.75, and γ = 0.125: HEA = -0.5776 SOC + 9.6333.**

**Figure S5. Social benefits (SOC) in [0, 10] vs. happiness (HAP) in [0, 10] for mathematicians and physicists if the weights are α = 0.125, β = 0.75, and γ = 0.125: HAP = -0.062 SOC + 7.4674.**

**Figure S6. Social benefits (SOC) in [0, 10] vs. health (HEA) in [0, 10] for mathematicians and physicists if the weights are α = 0.125, β = 0.75, and γ = 0.125: HEA = -0.0992 SOC + 9.2763.**

**Figure S7. Social benefits (SOC) in [0, 10] vs. happiness (HAP) in [0, 10] for biologists and chemists if the weights are α = 0.125, β = 0.75, and γ = 0.125: HAP = -0.0914 SOC + 7.4662.**

**Figure S8. Social benefits (SOC) in [0, 10] vs. health (HEA) in [0, 10] for biologists and chemists if the weights are α = 0.125, β = 0.75, and γ = 0.125: HEA = -0.1072 SOC + 9.3556.**

**Figure S9. Social benefits (SOC) in [0, 10] vs. the number of creators (*N*) in [0, 50] for music composers: SOC = -2.319 ln(N) + 9.395.**

**Figure S10. Social benefits (SOC) in [0, 10] vs. the number of creators (*N*) in [0, 50] for painters: SOC = -2.038 ln(N) + 8.2634.**

**Figure S11. Social benefits (SOC) in [0, 10] vs. the number of creators (*N*) in [0, 50] for mathematicians and physicists: SOC = -3.193 ln(N) + 12.005.**

**Figure S12. Social benefits (SOC) in [0, 10] vs. the number of creators (*N*) in [0, 50] for biologists and chemists: SOC = -2.826 ln(N) + 9.7929.**
